# Identification and Characterization of Genes Related to Ampicillin Antibiotic Resistance in *Zymomonas mobilis*

**DOI:** 10.3390/antibiotics11111476

**Published:** 2022-10-25

**Authors:** Binan Geng, Xingyu Huang, Yalun Wu, Qiaoning He, Shihui Yang

**Affiliations:** State Key Laboratory of Biocatalysis and Enzyme Engineering, Environmental Microbial Technology Center of Hubei Province, School of Life Sciences, Hubei University, Wuhan 430062, China

**Keywords:** *Zymomonas mobilis*, antibiotic resistance, ampicillin, genome editing, CRISPR−Cas12a, resistance selection markers

## Abstract

Antibiotics can inhibit or kill microorganisms, while microorganisms have evolved antibiotic resistance strategies to survive antibiotics. *Zymomonas mobilis* is an ideal industrial microbial chassis and can tolerate multiple antibiotics. However, the mechanisms of antibiotic resistance and genes associated with antibiotic resistance have not been fully analyzed and characterized. In this study, we investigated genes associated with antibiotic resistance using bioinformatic approaches and examined genes associated with ampicillin resistance using CRISPR/Cas12a−based genome−editing technology. Six ampicillin−resistant genes (*ZMO0103*, *ZMO0893*, *ZMO1094*, *ZMO1650*, *ZMO1866*, and *ZMO1967*) were identified, and five mutant strains ZM4∆0103, ZM4∆0893, ZM4∆1094, ZM4∆1650, and ZM4∆1866 were constructed. Additionally, a four−gene mutant ZM4∆ARs was constructed by knocking out *ZMO0103*, *ZMO0893*, *ZMO1094*, and *ZMO1650* continuously. Cell growth, morphology, and transformation efficiency of mutant strains were examined. Our results show that the cell growth of ZM4∆0103 and ZM4∆ARs was significantly inhibited with 150 μg/mL ampicillin, and cells changed to a long filament shape from a short rod shape. Moreover, the transformation efficiencies of ZM4∆0103 and ZM4∆ARs were decreased. Our results indicate that ZMO0103 is the key to ampicillin resistance in *Z. mobilis*, and other ampicillin−resistant genes may have a synergetic effect with it. In summary, this study identified and characterized genes related to ampicillin resistance in *Z. mobilis* and laid a foundation for further study of other antibiotic resistance mechanisms.

## 1. Introduction

Antibiotics are natural secondary metabolites or artificially synthesized analogs produced by organisms, such as bacteria, animals, and plants during the metabolic process to kill pathogens [1]. They are commonly used during plasmid and strain construction in genetic engineering and are usually used as the feed additive for the growth and disease resistance of plants and animals [2]. In the environment, competition exists among microorganisms for living space and nutrients. Some microorganisms compete with others by producing antibiotics that are sensitive to antibiotics. For example, *streptomyces* can produce 80% of the antibiotics currently known [3]. At the same time, various microorganisms naturally have certain resistance to different antibiotics, which brings challenges to the genetic engineering of strains [4].

At present, four classes of antibiotics are mainly classified according to their inhibition pathway: (i) inhibition of cell wall synthesis, such as β−lactam antibiotics and glycopeptides; (ii) inhibition of protein synthesis including macrolides, oxazolidinones, amphenicols, lincosamides, tetracyclines, and aminoglycosides [5]; (iii) inhibition of DNA synthesis by targeting gyrase or DNA, such as fluoroquinolones and nitroimidazoles [6,7]; and (iv) inhibition of membrane integrity, such as lipopeptides (e.g., daptomycin) [8] and polymyxins (e.g., colistin) [9]. β−Lactams are the most widely used antibiotics with the potential to interrupt bacterial cell wall formation as a result of covalent binding to essential penicillin−binding protein (PBP) enzymes that are involved in the terminal steps of peptidoglycan cross−linking in both Gram−negative and Gram−positive bacteria [10]. Ampicillin is the representative β−lactam antibiotic and commonly used in genetic engineering to screen colonies with ampicillin resistance.

To survive in an environment containing antibiotics, microorganisms have developed a variety of antibiotic resistance (AR) mechanisms during evolution. Based on the biochemical route involved in resistance, the mechanisms of antibiotic resistance currently are classified into four types. The first one is to modify the antimicrobial molecule. Some enzymes are capable of chemical alterations to inactivate the antibiotics with acetylation, phosphorylation, and adenylation of the antibiotics, such as aminoglycosides, chloramphenicol, and streptogramins [11]. Some enzymes can destroy the antibiotic molecule, such as β−lactamases, rendering the antibiotic unable to interact with the target sites [11]. The second is to prevent antibiotics from reaching their target by decreasing penetration or actively extruding the antimicrobial compound. A reduced number or differential expression of porins, such as the OprD porin protein in some microorganisms, prevented the entry of carbapenems [12]. Extruding the toxic compound out of the cell through an efflux pump [13,14] and the formation of biofilm [15] are also effective to prevent the entrance of antibiotics. The third one is to change or bypass target sites by avoiding the antibiotic to reach its binding site or to modify the target sites that results in decreased affinity for the antibiotic molecule. The last type is a global cell adaptive response to the antibacterial attack.

*Z. mobilis* is a facultative anaerobic Gram−negative bacterium with a unique Entner–Doudoroff (ED) pathway and many excellent physiological characteristics for industrial bioethanol production, such as the highly efficient utilization of sugar and high ethanol yield and ethanol tolerance [16,17]. Currently, the available antibiotics used for genetic engineering in *Z. mobilis* include ampicillin, kanamycin, spectinomycin, chloramphenicol, tetracycline, streptomycin, and gentamicin [18,19]. Among them, some antibiotics are naturally resisted by *Z. mobilis* at low concentrations, such as ampicillin < 300 μg/mL, kanamycin < 350 μg/mL, streptomycin < 300 μg/mL, gentamicin < 100 μg/mL, and tetracycline and chloramphenicol < 25 μg/mL [18,19]. In addition, different subspecies of *Z. mobilis* have variable susceptibility to different antibiotics. For example, the working concentrations of ampicillin, chloramphenicol, tetracycline, and kanamycin that are used for genetics studies were 300 vs. 500, 100 vs. 100, 25 vs. 25, and 350 vs. 250 μg/mL in *Z. mobilis* ZM4 and CP4, respectively [19]. 

Although *Z. mobilis* is tolerant to ampicillin, only one work reported that *ZMO0103* probably is an ampicillin−resistant gene, which reported the results of a heterologous protein expression and an enzymatic kinetic analysis [20]. The genome sequence of *Z. mobilis* ZM4 was published, and the genome annotation was further improved [21,22,23]. Moreover, the genome−editing tools including the native type I−F CRISPR−Cas system and the CRISPR−Cas12 system as well as the platform to identify and characterize biological parts have been established in *Z. mobilis* [24,25,26]. Therefore, we attempted to explore the ampicillin tolerance mechanism of *Z. mobilis* by identifying potential resistance genes using bioinformatics approaches and constructing ampicillin−sensitive mutant strains by genome engineering to verify its function. 

## 2. Results

### 2.1. In Silico Analysis of the AR Genes of Z. mobilis ZM4

A total of 100 candidate AR genes in *Z. mobilis* ZM4 were predicted using the databases of CARD and MEGARes. The results demonstrate that *Z. mobilis* ZM4 contains 9 putative lactamase−related genes (*ZMO0103*, *ZMO0108*, *ZMO0598*, *ZMO0675*, *ZMO0781*, *ZMO1336*, *ZMO1574*, *ZMO1914*, and *ZMO1967*), 7 putative transferase−related genes (*ZMO0111*, *ZMO0183*, *ZMO1143*, *ZMO1306*, *ZMO1355*, *ZMO1452*, and *ZMO1577*), 8 putative porin−related genes (*ZMO0079*, *ZMO0257*, *ZMO0478*, *ZMO1124*, *ZMO1164*, *ZMO1177*, *ZMO1322*, and *ZMO1387*), and 76 putative efflux pump−related genes (See Supplementary Materials Table S1). In addition, 68 β−lactamase genes were predicted in ZM4 by BLASTP with the gene sequences of the β−lactam class in the UniProt database (See Supplementary Materials Table S2). Among them, six genes were annotated as β−lactamase−encoding genes (*ZMO0103*, *ZMO0893*, *ZMO1094*, *ZMO1650*, *ZMO1866*, *ZMO1967*), two candidate genes in list 1 (*ZMO0103*, *ZMO1967*), and five genes in list 2 (*ZMO0103*, *ZMO0893*, *ZMO1650*, *ZMO1866*, and *ZMO1967*). 

Five putative ampicillin−resistant (AR) candidate genes of *ZMO0103*, *ZMO0893*, *ZMO1650*, *ZMO1866*, and *ZMO1967* were predicted as the β−lactamase genes, and *ZMO1094* was annotated as metallo−beta−lactamase−like protein−encoding gene in ZM4. Multiple sequence alignment revealed that ZMO0103, ZMO0893, and ZMO1650 belong to the AmpC superfamily (Supplementary Materials Figure S1); ZMO1967 and ZMO1094 belong to the PenP superfamily (β−lactamase class A), while ZMO1866 belongs to the RnjA superfamily (Supplementary Materials Figure S1) according to the conserved domain search [27]. Moreover, ZMO0103, ZMO0893, and ZMO1650 have similar conserved structures based on the multisequence alignment results (Supplementary Materials Figure S2).

### 2.2. Ampicillin Resistance−Related Gene Knockout in Z. mobilis ZM4

**Figure 1:**
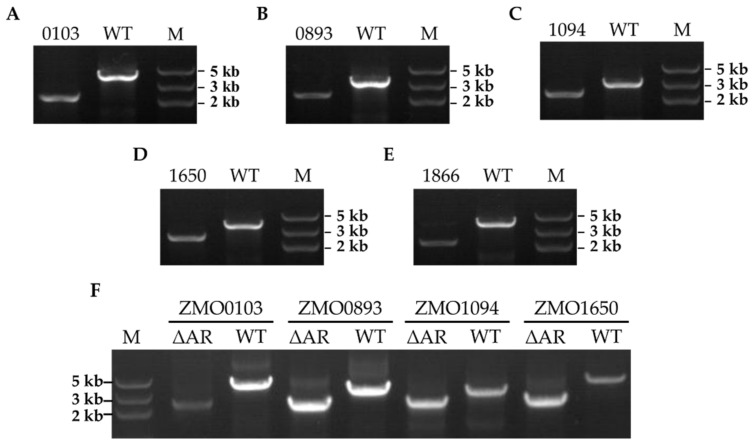
Confirmation of ampicillin−resistant (AR) knockout strains in *Z. mobilis* ZM4 by PCR. The mutants of ZM4∆0103 (**A**), ZM4∆0893 (**B**), ZM4∆1650 (**C**), ZM4∆1094 (**D**), ZM4∆1866 (**E**), and ZM4∆ARs (**F**) were confirmed by colony PCR using their corresponding primers. The sizes of PCR products (bp) of WT and knockout strains were 4021, 2247 (ZM4∆0103); 3314, 2232 (ZM4∆0893); 3122, 2361 (ZM4∆1650); 3746, 2221 (ZM4∆1094); 3768, 2080 (ZM4∆1866).

Subsequently, six ampicillin−resistant (AR) candidate genes based on the above bioinformatics study were selected for knockout using the CRISPR−Cas12a genome−editing system. Except for *ZMO1967,* which may be an essential gene and cannot be knocked out, five other genes of *ZMO0103*, *ZMO0893*, *ZMO1094*, *ZMO1650*, and *ZMO1866* were successfully knocked out with the CRISPR−Cas12a system. As demonstrated in Figure 1, the expected sizes of the amplicon of *ZMO0103*, *ZMO0893*, *ZMO1094*, *ZMO1650*, and *ZMO1866* in the wild type (WT) strain were obtained, which were ~4021, 3314, 3122, 3746, and 3768 bp, respectively. As a contrast, the corresponding amplified fragments in the knockout strains were ~2247, 2232, 2361, 2221, and 2080 bp, respectively (Figure 1). The inactivation of the AR genes was further confirmed by Sanger sequencing of the PCR products. Then, the knockout strains harboring the editing plasmids were cultured in RMG5 for several passages to obtain the final strains with the editing plasmid cured. Except for *ZMO1866*, *ZMO0103*, *ZMO0893*, *ZMO1094,* and *ZMO1650,* the knockout strains lost the editing plasmid successfully, which can only grow in RMG5 after 24 h cultivation, but not in the medium with chloramphenicol (Supplementary Materials Figure S2).

To obtain the strain with all the ampicillin−resistant (AR) candidate genes knocked out, we further conducted six rounds of genome editing continuously with the CRISPR−Cas12a system. Consistent with the above single−gene deletion experiments, we only obtained a mutant strain, ZM4∆Ars, with four ampicillin−resistant (AR) genes of *ZMO0103*, *ZMO0893*, *ZMO1094,* and *ZMO1650* knocked out continuously, while ZMO1967 and ZMO1866 were not able to be deleted. The ZM4∆ARs was further identified by colony PCR using the primers for each gene. The results of the correct PCR products of four genes indicate that these four ampicillin−resistant (AR) genes were knocked out successfully in ZM4∆ARs (Figure 1F).

### 2.3. Antibiotic Tolerance of Ampicillin−Resistant (AR) Gene Knockout Strains 

**Figure 2:**
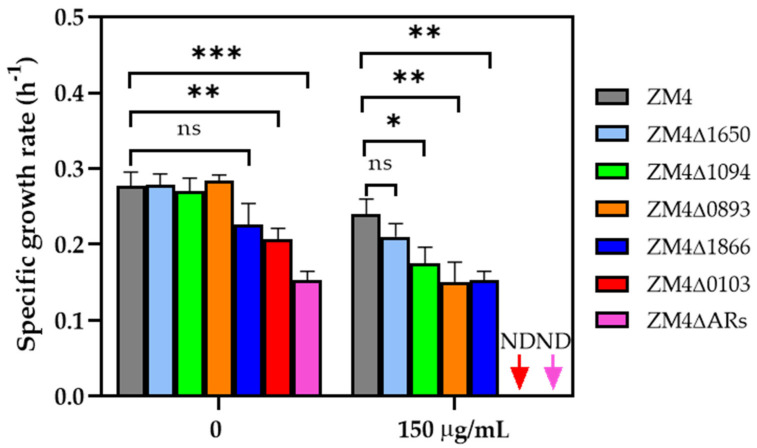
The specific growth rate of ampicillin−resistant (AR) gene knockout strains cultured under 0 and 150 μg/mL of ampicillin. Three replicates were performed for the experiment. When the mutant could not grow under the condition, the sample is marked “ND” (not detected). * represents a significant difference with *p*−value < 0.05. ** represents a significant difference with *p*−value < 0.01. *** represents a significant difference with *p*−value < 0.001. ns represents no significant difference.

Previous studies reported that the concentration of ampicillin required for plate screening and liquid culture of the transformants in genetic engineering manipulation of *Z. mobilis* ZM4 was 300 μg/mL [19]. In this study, we set three ampicillin concentration gradients of 0, 150, and 300 μg/mL to cultivate the ampicillin−resistant (AR) gene knockout strains. As demonstrated in the Supplementary Materials Figure S4, the deletion of *ZMO1094* and *ZMO1650* individually did not decrease the ampicillin resistance. Specifically, the growth of ZM4∆1094 and ZM4∆1650 under different ampicillin concentrations was not inhibited compared with ZM4, whereas the deletion of *ZMO0893* and *ZMO1866* individually were effective. The growth of ZM4∆0893 and ZM4∆1866 was inhibited under 150 μg/mL ampicillin with a slower growth rate of 0.15 ± 0.03 h^−1^ and 0.05 ± 0.01 h^−1^ compared with the value of 0.24 ± 0.02 h^−1^ for wild−type ZM4 (Figure 2). The growth of the *ZMO0103* mutant ZM4∆0103 was the slowest among all strains with the lowest growth rate of 0.21 ± 0.01 h^−1^ under 0 μg/mL ampicillin, and ZM4∆0103 was inhibited by all concentrations of ampicillin, such as 150 μg/mL (Figure 2, Supplementary Materials Figure S4). 

The same experiment was also carried out for ZM4∆ARs, a mutant strain with four ampicillin−resistant (AR) genes deleted. Under 0 μg/mL ampicillin, a long lag phase was observed with a lower growth rate of 0.15 ± 0.01 h^−1^ in ZM4∆ARs compared to ZM4∆0103 (Figure 2, Supplementary Materials Figure S4), and ZM4∆ARs also cannot grow at 150 μg/mL ampicillin. Combined with the growth of single−gene knockout strains under RMG5, we speculated that the deletion of *ZMO0103* is one of the major reasons for the poor growth of ZM4∆ARs, and other ampicillin−resistant (AR) genes may have a synergetic effect on ampicillin resistance. However, as previously reported, when the ampicillin concentration increased to 300 μg/mL, nearly all the strains could not grow. These results also demonstrate that the mutants ZM4∆0103 and ZM4∆ARs had a lower ampicillin tolerance concentration—150 μg/mL.

### 2.4. Cell Morphology of Mutants Treated with Ampicillin

**Figure 3:**
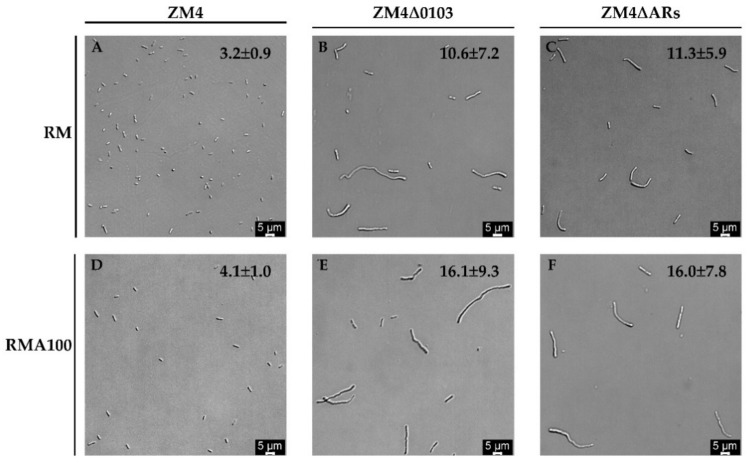
Cell morphology of strains ZM4, ZM4∆0103, and ZM4∆ARs cultured in RM and RMA100 was observed by light microscopy. The numbers with error value in each image represent the average cell size (μm) analyzed with ImageJ software. Numbers in the lower right corner of each represent the scale. RM and RMA100 represent the different RMG5 media with 0 and 100 μg/mL of ampicillin.

Previous studies demonstrated that cell cultures in the presence of antibiotics resulted in abnormal cellular morphology in various degrees, such as elongated or distorted cell shapes [28,29]. To evaluate the morphological changes of ampicillin−resistant (AR) gene knockout strains during the ampicillin treatment, cell morphologies of three *Z. mobilis* strains (ZM4, ZM4∆0103, and ZM4∆Ars) were observed under light microscopy. The results show that ZM4∆0103 and ZM4∆ARs had longer rod shapes with various cell lengths and widths. The lengths of ZM4∆0103 and ZM4∆ARs were around 10.6 ± 7.2 μm and 11.3 ± 5.9 μm compared to that of wild−type ZM4 (3.2 ± 0.9 μm) in RMG5 without ampicillin (Figure 3). However, when strains were cultured in RMG5 with 100 μg/mL ampicillin (RMA100), the cellular morphology of ZM4∆0103 (16.1 ± 9.3 μm) and ZM4∆ARs (16.0 ± 7.8 μm) changed and was longer with a filament shape, while the length of ZM4 did not change much (4.1 ± 1.0 μm). This demonstrated that ampicillin was more stressful to ZM4∆0103 and ZM4∆ARs during cell growth, and ZM4∆0103 and ZM4∆ARs were more sensitive to ampicillin than ZM4. Similar morphological changes of *Z. mobilis* have been previously described when the cells were exposed to different stresses, such as high temperature [30], lignocellulosic hydrolysate inhibitory [31], high concentration of xylose [32], and salt conditions [33].

### 2.5. Effects of Ampicillin−Resistant (AR) Gene Mutagenesis on Genetic Transformation Efficiency

**Figure 4:**
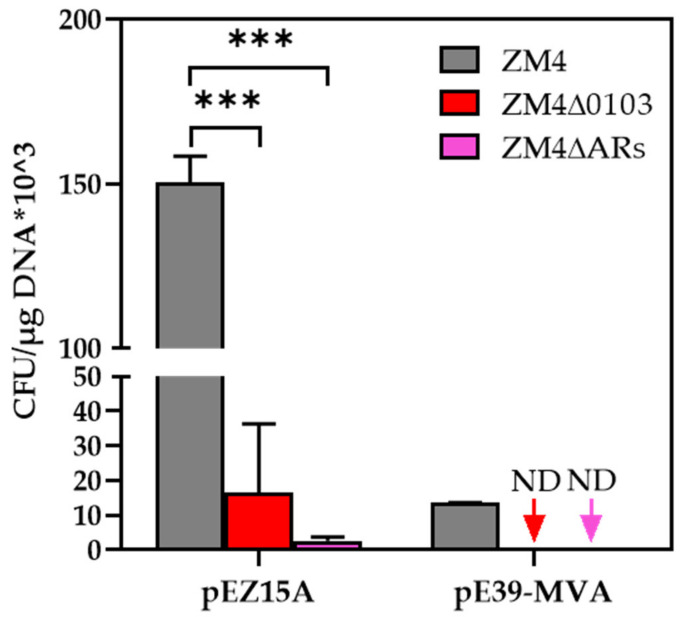
Electroporation efficiency of ZM4, ZM4∆0103, and ZM4∆ARs using plasmids of pEZ15A (~3 kb) and pE39−MVA (~10 kb). Three replicates were performed for the experiment. The error bar represents standard deviation (SD). When transformation of a plasmid was below the limit of detection (0.00001), the sample is marked “ND” (not detected). *** represents a very significant difference (*p*−value < 0.001).

The transformation efficiencies of both ZM4∆0103 and ZM4∆ARs were significantly reduced using the plasmids of pEZ15A (~3 kb) and pE39−MVA (~10 kb). The electroporation efficiency of plasmid pEZ15A in ZM4 was (1.50 ± 0.08) × 10^5^ CFU/μg DNA, which was decreased to (1.66 ± 1.98) × 10^4^ and (2.66 ± 1.11) × 10^3^ CFU/μg DNA in ZM4∆0103 and ZM4∆ARs, respectively (Figure 4, Supplementary Materials Figure S5), with the transformation efficiencies reduced ca 10~100 fold. Few colonies grew on the plate after the electroporation of pE39−MVA to ZM4∆0103 and ZM4∆ARs, while the efficiency of (1.35 ± 0.01) × 10^4^ CFU/μg DNA in ZM4 was observed (Figure 4, Supplementary Materials Figure S6). The transformation efficiency of ZM4∆0103 and ZM4∆ARs cannot be calculated when the pE39−MVA plasmid with a large size was used. These results suggest that the predicted β−lactamase genes influenced the genetic transformation efficiency and significantly reduced the electroporation efficiency especially with the larger plasmid.

## 3. Discussion

In this study, we first predicted six functional β−lactamase genes that may cause ampicillin antibiotic resistance in *Z. mobilis* ZM4 by bioinformatics analysis. ZMO0103, ZMO0893, and ZMO1650 belong to the AmpC superfamily containing similar conserved structures, indicating that they may have similar functions in cellular processes. ZMO1967 belongs to the PenP superfamily, which is associated with β−lactamase class A. ZMO1094 and ZMO1866 are not closely related to the function of β−lactams. We attempted to knock out all six of them in ZM4; unfortunately, only 5 of them were deleted individually using the CRISPR−Cas12a genome−editing system. The ZMO1967 protein has a transmembrane domain at the N−terminus with a probability of 0.98 by TMHMM−2.0 (https://dtu.biolib.com/DeepTMHMM, accessed on 24 August 2022). In addition, *ZMO1967* is probably an essential gene according to the results of the genome−wide CRISPRi (unpublished data), which may be the reason that it failed to be knocked out in this study. We successfully deleted five β−lactamase genes individually in ZM4, but the editing plasmid for *ZMO1866* deletion was not able to be eliminated.

Our study demonstrated that in addition to ampicillin resistance, these genes annotated as ampicillin−resistant (AR) genes had other effects on cell morphology, cell growth, and transformation efficiency. The knockouts *ZMO0893*, *ZMO1094*, and *ZMO1866* slightly inhibited the growth under the addition of 150 μg/mL. Especially, when *ZMO0103* was deleted, the growth of the strain was inhibited mostly with different concentrations of ampicillin. The biomass of ZM4∆0103 hardly increased under ampicillin ≥ 150 μg/mL. In addition, similar growth inhibition was also observed in four ampicillin−resistant (AR) genes of *ZMO0103*, *ZMO0893*, *ZMO1094* and the *ZMO1650* knockout strain ZM4∆ARs. The previous study reported that *ZMO0103* was a β−lactamase gene, which contains a 55% amino acid sequence identity with class C β−lactamase genes [20]. A higher expression level [30,34,35,36] of *ZMO0103* in ZM4 may be ascribed to the absence of the AmpR that can inhibit the expression of *ZMO0103*. The result of multiple sequence alignment shows that the homologous protein of β−lactamase ZMO0103 was only found in *Sphingomonas* (Supplementary Materials Figures S7 and S8). Combining the result of the improved sensitivity of ZM4∆0103 to ampicillin in this study, we speculated that ZMO0103 is a unique protein and the most important β−lactamase in *Z. mobilis* ZM4, resulting in the high resistance of ZM4 to β−lactam antibiotics, such as ampicillin [17].

In addition, we also found that the growth of ZM4∆0103 and ZM4∆ARs was inhibited even without the addition of ampicillin. Considering the existence of transmembrane helices and signal peptides at the N−terminus (https://services.healthtech.dtu.dk/service.php?SignalP, accessed on 28 August 2022) of ZMO0103, ZMO0103 located on the cell membrane may directly hydrolyze ampicillin in the periplasmic space, and the integrity of the membrane could be disrupted by knocking out *ZMO0103* leading to the defective growth of ZM4∆0103 and ZM4∆ARs. Furthermore, cell sizes of ZM4∆0103 and ZM4∆ARs became longer and further lengthened with the addition of ampicillin indicating that the AmpC family lactamase protein ZMO0103 is related to cell wall biosynthesis and deconstruction and crucial for cell morphology and growth. Microscopic observations under RMG5 showed that the longest cells in ZM4∆0103 and ZM4∆ARs increased in length by 13.7 μm and 13.1 μm, respectively, compared to the longest cells in the wild−type ZM4. In addition, when 100 μg/mL of ampicillin (RMA100) was added, the cell lengths of the longest cells in ZM4∆0103 and ZM4∆ARs could increase by 7.6 μm and 6.6 μm, respectively, compared to the lengths of ZM4∆0103 and ZM4∆ARs under RM. These results suggest that the deletion of ZMO0103 affected the cell wall structure and the cell membrane and therefore led to changes in the intracellular osmotic pressure, thus enlarging the cells similar to the result of the previous study [37].

Based on our speculation that the deletion of *ZMO0103* may affect cell membrane and cell wall structures, we expected that the exogenous DNA could be more easily transferred into the cells. So, we further tested the transform efficiency of ZM4∆0103 and ZM4∆ARs using two plasmids of pEZ15A and pE39−MVA with different sizes. However, lower transformation efficiencies were observed when pEZ15A and pE39−MVA were electroporated into ZM4∆0103 and ZM4∆ARs compared with ZM4. Particularly, in the case of pE39−MVA, almost no transformants were obtained, which suggested that the transformation efficiency of the knockout strains was affected by plasmids sizes. However, the mechanisms of how *ZMO0103* influences the transformation efficiency remain to be investigated including the constructing a truncated mutant of *ZMO0103* by deleting the catalytic domain (amino acids from position 52 to 405) or the C−terminal domain (amino acids from position 406 to 520) that may influence the transformation of exogenous DNA. In addition, the restriction–modification (R–M) system genes (*ZMO0028*, *ZMO1933*, and *ZMOp32x025_028*) could be knocked out in ampicillin−resistant gene knockout strains to further improve the transformation efficiency [38,39,40].

## 4. Materials and Methods

### 4.1. Strains and Cultural Conditions

*E. coli* DH5α was stored in our laboratory and used for plasmid maintenance and construction. During culturing, 50 μg/mL of chloramphenicol was added to Luria−Bertani (LB) medium (10 g/L tryptone, 5 g/L yeast extract, and 10 g/L NaCl, pH 7.0) for *E. coli* and cultured at 37 °C. *Z. mobilis* ZM4 was used as the parental strain for the construction of derived mutants and cultured with Rich Medium (RMG5) (50 g/L glucose, 10 g/L yeast extract, and 2 g/L KH_2_PO_4_) at 30 °C. When required, 50 μg/mL of chloramphenicol and 100 μg/mL of spectinomycin were added to the LB and RMG5. All *E. coli*, *Z. mobilis,* and their derivative strains used in this study are listed in the Supplementary Materials Table S3.

### 4.2. In Silico Analysis of the AR Genes of Z. mobilis ZM4

A BLASTP analysis of the *Z. mobilis* ZM4 proteome was performed using two databases for resistance genes: CARD (Comprehensive antibiotic resistance database) and MEGARes database. In addition to using BLASTP to investigate the relatedness of the sequences to those contained within the CARD and MEGARes databases, we further aligned with the gene sequences of the β−lactamase class in the UniProt database with the *Z. mobilis* ZM4 protein sequences. We chose the common genes of candidate β−lactamase genes after BLASTP with CARD and MEGARes databases and genes annotated as β−lactamase as candidate gene list 1. Then, we chose the common genes of candidate β−lactamase genes after BLASTP with the UniProt database and genes annotated as β−lactamase as candidate gene list 2. The common genes within both candidate gene list 1 and list 2 were the final candidate β−lactamase genes used for investigation in this work. The β−lactamase genes with a significant E−value of 2 × 10^−10^ were selected.

### 4.3. Construction of Editing Plasmids of Ampicillin−Resistant (AR) Genes in Z. mobilis ZM4

The spacers were designed to bear the entire 23 bp sequences containing a 5′−NTTN−3′ PAM for six genes of *ZMO0103*, *ZMO0893*, *ZMO1094*, *ZMO1358*, *ZMO1866,* and *ZMO1967*. The oligonucleotides of spacers were ordered from TsingKe Biotechnology Co., Ltd. (Beijing, China). CRISPR−Cas12a−editing plasmids were constructed following the previous description [25]. The Cas12a−targeting gRNA sequence was annealed using two single−stranded oligonucleotides by first heating the reaction mixture to 95 °C for 5 min and subsequently cooling down gradually to room temperature. Then, the annealed spacer was ligated into *Bsa* I−linearized pEZ−sgr by T4 ligase at 22 °C for 3 h. The resulting plasmids were named as pEZ−sgr−0103, pEZ−sgr−0893, pEZ−sgr−1094, pEZ−sgr−1385, pEZ−sgr−1866, and pEZ−sgr−1967.

Gibson assembly method was utilized for donor construction. Donor sequences including extra ~800 bp upstream and downstream flank sequences of the candidate gene were amplified using Primer STAR polymerase (Takara, Japan) from the genomic DNA of *Z. mobilis* ZM4 and then cloned into pEZ−sgr vector by T5 exonuclease (NEB, WA, USA). The resulting plasmids were named as pEZ−sgr−0103−D, pEZ−sgr−0893−D, pEZ−sgr−1094−D, pEZ−sgr−1385−D, pEZ−sgr−1866−D, and pEZ−sgr−1967−D. All plasmids used in this work are provided in the Supplementary Materials Table S4. The sequences of primers used in this study are shown in the Supplementary Materials Table S5.

### 4.4. Curing of Editing Plasmids

Transformants harboring editing plasmids were cultured in RMG5 broth without the supplement of antibiotics. After 6 consecutive passages in the nonresistant RMG5 liquid medium, 100 μL cultures were diluted and plated on nonresistant RMG5 plates. Then, single colonies were picked to conduct colony PCR using primers pEZ15A−F/R for amplifying the editing plasmid. Editing plasmids were lost if no PCR product was amplified compared with the control. Single colonies with correct PCR results were then inoculated on RMG5 with or without chloramphenicol for further verification. The knockout strains losing the editing plasmids can only grow in RMG5, but not in the medium with the supplementation of chloramphenicol.

### 4.5. Electroporation of Editing Plasmids to Z. mobilis ZM4

Transformation of *Z. mobilis* ZM4 with the editing plasmid was achieved by preparing electrocompetent cells using a modified protocol as previously described. *Z. mobilis* ZM4 was inoculated with 40 mL RMG5 in a 100 mL flask and was grown to an OD600 nm = 0.4~0.6. Cells were harvested by centrifugation at 4000× *g* for 10 min at room temperature. The supernatant was discarded, and the cell pellet was washed with Milli−Q^®^ ultrapure water and 10% (*v*/*v*) glycerol before being resuspended in a final volume of 400 μL of 10% (*v*/*v*) glycerol. Cells were stored as 50 μL aliquots on ice for immediate use in electroporation experiments. Then, 500 ng plasmid DNAs was used for electroporation (Bio−Rad, CA, USA). After pulsing at 16 kV/cm, 25 μF and 200 Ω, 1 mL of RMG5 was added to the electroporated solutions and then incubated at 30 °C for 4~6 h. Finally, the cells were plated and selected on RM plates supplemented with chloramphenicol until colonies were visible (≤2 d).

### 4.6. Construction of ZM4∆ARs by Continuous Gene Editing

We constructed ZM4∆ARs with four genes of *ZMO0103*, *ZMO0893*, *ZMO1094,* and *ZMO1650* knocked out following these steps:

Step 1. A CRISPR−Cas12a−editing plasmid pEZ−sgr−0103−D was transformed into ZM4−Cas12a. Colony PCR was then performed with the primers 0103−out−F and 0103−out−R.

Step 2. The positive single clone with the correct PCR size was cultured in RMG5, and the editing plasmid was then cured following Section 4.4.

Step 3. After curing the editing plasmid, the electrocompetent cells of knockout strain ZM4∆0103 were then prepared for the next−round knockout.

Knockout of *ZMO0893*, *ZMO1094*, *ZMO1650*, *ZMO1866*, and *ZMO1967* followed the above steps 1 to 3. After each gene was edited and the corresponding editing plasmid was lost, the competent cells of the corresponding mutant were prepared for transferring to the next editing plasmid.

### 4.7. Genetic Transformation Efficiency of β−lactamase Mutants

To calculate the electroporation efficiencies of the β−lactamase mutants, a 3−kb shuttle plasmid pEZ15A and a 10−kb shuttle plasmid pE39−MVA were prepared from *E. coli* DH5α and introduced into ZM4, ZM4∆0103, and ZM4∆Ars. Electroporation efficiency was presented by the colony forming units (CFUs) on selective plates when 50 μg plasmid DNA was introduced and 100 μL recovery culture was plated. The calculating formula is described below:

CFU/μg^−1^ DNA = (Cp/Tp) × (Vt/Vp);

where Cp is the colony number counted on selective plates; Tp is the total amount of plasmid DNA (μg) used here; Vt is the total transformation volume (μL); Vp is the volume (μL) plated.

### 4.8. Confirmation of Ampicillin−Resistant Gene Deletion in Z. mobilis ZM4

A pair of primers was used to confirm the disruption of each AR gene in *Z. mobilis* transformants: the primers were used to amplify the region including upstream of the corresponding gene, targeting gene, and downstream of the corresponding gene (see primer pairs in the Supplementary Materials Table S5, e.g., 0103−out−F and 0103−out−R).

### 4.9. Growth Studies and Analysis of Ampicillin−Resistant Gene Mutants

Cell growth was monitored by measuring the cell optical density (OD) values using a Bioscreen C high−throughput growth measurement instrument (Bioscreen C MBR, Helsinki, Finland). The single colony was inoculated in 1 mL of RMG5 at 30 °C overnight as the seed culture. Then, the seed culture of *Z. mobilis* was transferred to RMG5 until reaching the exponential phase. Subsequently, the cultures were inoculated to the well of the Bioscreen C plate containing 300 μL bacterium suspension with an initial OD_600 nm_ value of 0.05. Three technical replicates were used for each condition.

### 4.10. Cell Morphology Observation

*Z. mobilis* strains were cultured in RMG5 or RMG5 with 150 μg/mL ampicillin overnight at 30 °C. Bacterial pellets were collected at the exponential phase, washed twice with 1× phosphate buffer saline (1× PBS), resuspended in the same buffer, and observed under a light microscope (Leica Dmi8, Buffalo Grove, IL, USA) at 400 magnifications. Each image was taken using the image−based autofocus system LAS X software of the Leica Dmi8 system, and the cell size was measured using ImageJ software [41].

## 5. Conclusions

In summary, genes associated with antibiotic resistance in *Z. mobilis* were systematically investigated, and six ampicillin−resistant genes in *Z. mobilis* were identified. Five of them were knocked out individually, and four ampicillin−resistant gene deletion mutant ZM4∆Ars was also constructed to verify their functions. The strains of ZM4∆0103 and ZM4∆Ars were sensitive to ampicillin at a lower concentration of 150 μg/mL with a long and filament shape. The putative membrane protein ZMO0103 is probably the essential β−lactamase, and other ampicillin−resistant genes may have a synergetic effect with it. This study not only identified the ampicillin−resistant genes in *Z. mobilis* ZM4, but also verified their functions on cell growth, morphology, and transformation efficiency. In addition, ampicillin−sensitive mutants can serve as the parental strains for metabolic engineering practices in *Z. mobilis* enabling the usage of antibiotics, such as ampicillin, that are routinely used for genetic engineering.

## Data Availability

No new data were created or analyzed in this study. Data sharing does not apply to this article.

## Supplementary Materials

The following supporting information can be downloaded at: https://www.mdpi.com/article/10.3390/antibiotics11111476/s1, Figure S1: Secondary structure prediction of ZMO0103, ZMO0893, ZMO1094, ZMO1650, ZMO1866, and ZMO1967 in NCBI database; Figure S2: Multiple sequence alignment of the AmpC superfamily and β−lactamase class A protein in Z. mobilis; Figure S3: Loss of editing plasmids in ampicillin−resistant (AR) gene knockout strains; Figure S4: Cell growth of single and multiple ampicillin−resistant gene knockout strains cultured under 0 (A), 150 (B), and 300 (C) μg/mL of ampicillin; Figure S5: Electroporation efficiency of ZM4∆0103, ZM4∆Ars, and ZM4 by pEZ15A; Figure S6: Electroporation efficiency of ZM4∆0103, ZM4∆Ars, and ZM4 by pEZ15A and pE39−MVA; Figure S7: Distance tree of ZMO0103 after multiple sequence alignment with other proteins collected in NCBI using BALSTP; Figure S8: The detailed result of multiple sequence alignment with other proteins collected in NCBI using BALSTP; Table S1: List of the putative efflux pump−type antibiotic−resistant genes by BLASTP with CARD and MEGARes databases; Table S2: List of the putative β−lactamase genes by BLASTP in the UniProt database; Table S3: Strains used in this study; Table S4: Plasmids used in this study; Table S5: Primers used in this study.
