# Supplementary material for: Identification and Characterization of Genes Related to Ampicillin Antibiotic Resistance in Zymomonas mobilis
Source: Antibiotics (Basel). 2022 Oct 25;11(11):1476. doi: 10.3390/antibiotics11111476 (PMC9686808; doi:10.3390/antibiotics11111476)
Supplement: Supplementary file 1 [file antibiotics-11-01476-s001.zip › antibiotics-1936628-supplementary.pdf]

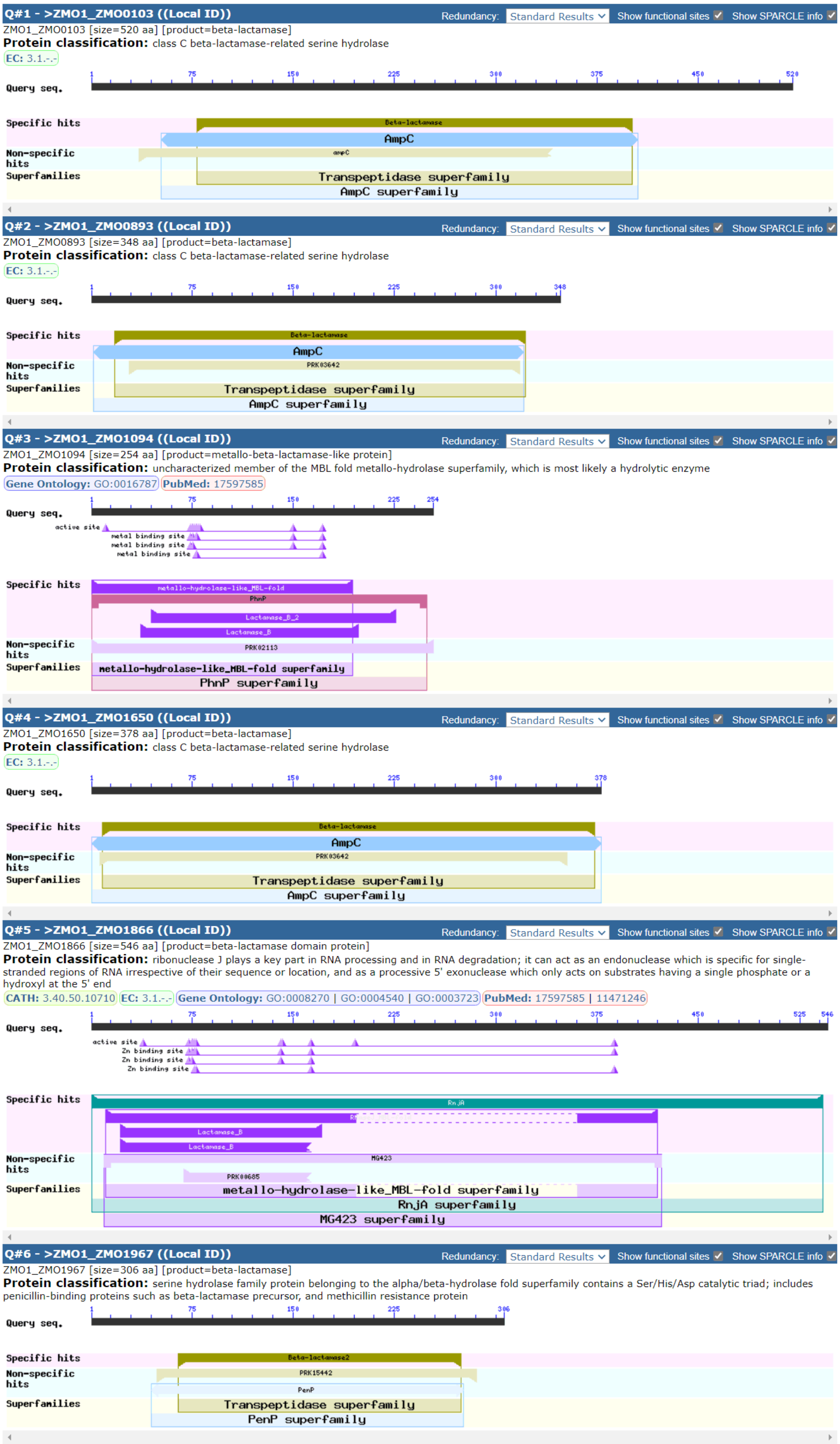

Figure S1. Secondary structure prediction of ZMO0103, ZMO0893, ZMO1094, ZMO1650, ZMO1866 and ZMO1967 in NCBI database.

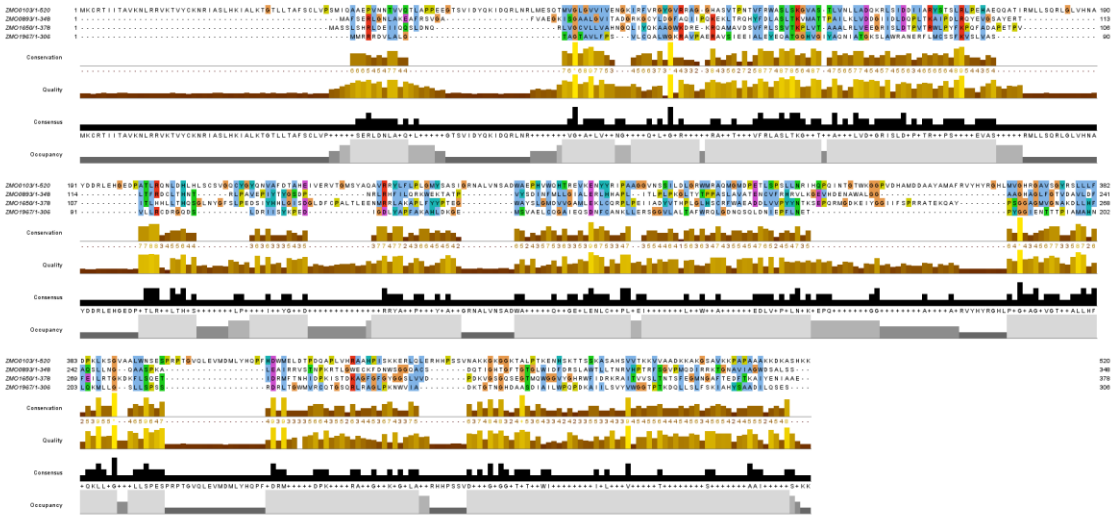

Figure S2. Multiple sequence alignment of the AmpC superfamily and β-lactamase class A protein in *Z. mobilis*.

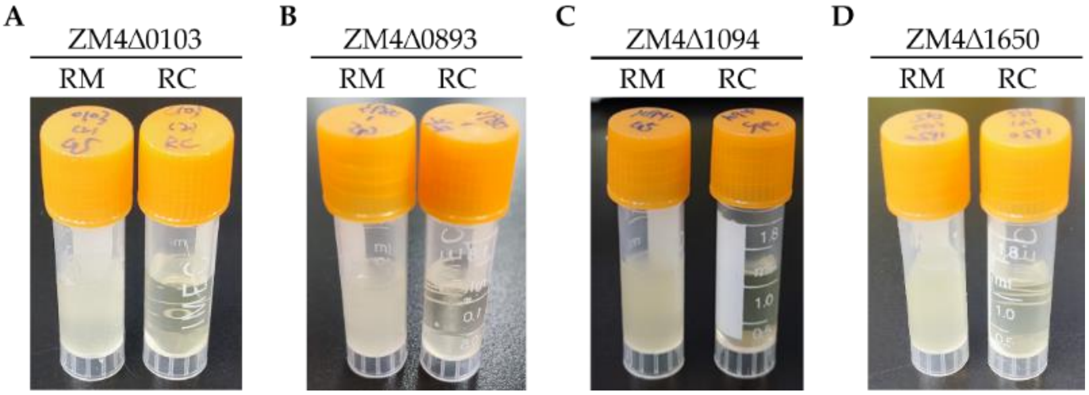

Figure S3. Loss of editing plasmids in ampicillin-AR gene knockout strains. Single colony of ZM4Δ0103 (A), ZM4Δ0893 (B), ZM4Δ1094 (C) and ZM4Δ1650 (D) without editing-plasmid verified by colony PCR was cultured in RMG5 (left) and RMG5 supplemented with 50 μg/mL of chloramphenicol (right), respectively.

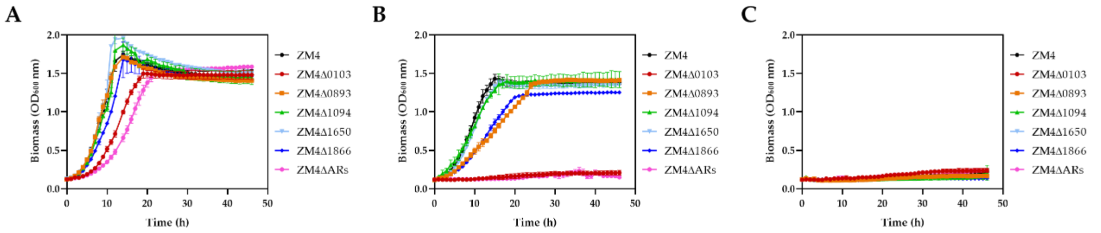

Figure S4. Cell growth of single and multiple ampicillin-resistance gene knockout strains cultured under 0 (A), 150 (B), and 300 (C) μg/mL of ampicillin, respectively.

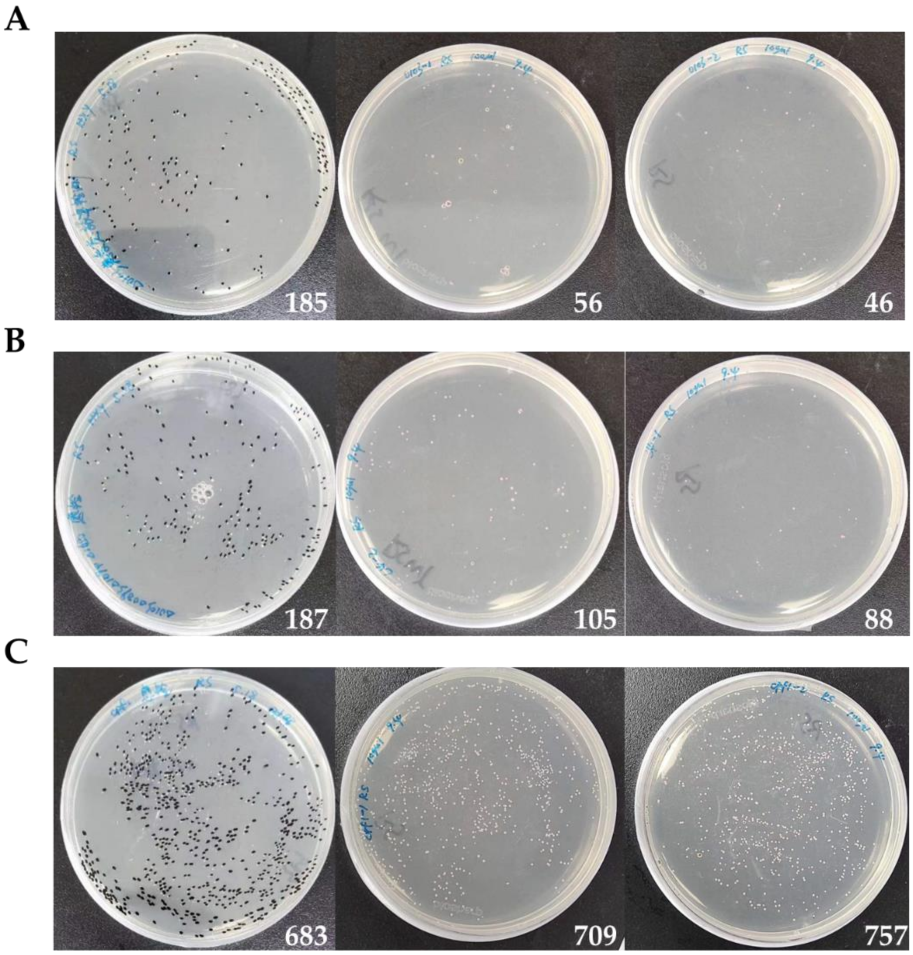

Figure S5. Electroporation efficiency of ZM4Δ0103, ZM4ΔARs and ZM4 by pEZ15A. Electroporation efficiency was tested by transforming pEZ15A into ZM4Δ0103 (A) and ZM4ΔARs (B), whereas transformed into ZM4 (C) as control. An equal volume of transformants was spread onto RM plates containing to 100 μg/mL spectinomycin. Numbers in the lower right corner of each plate represent numbers of colonies. Three replicates were performed for each Electroporation.

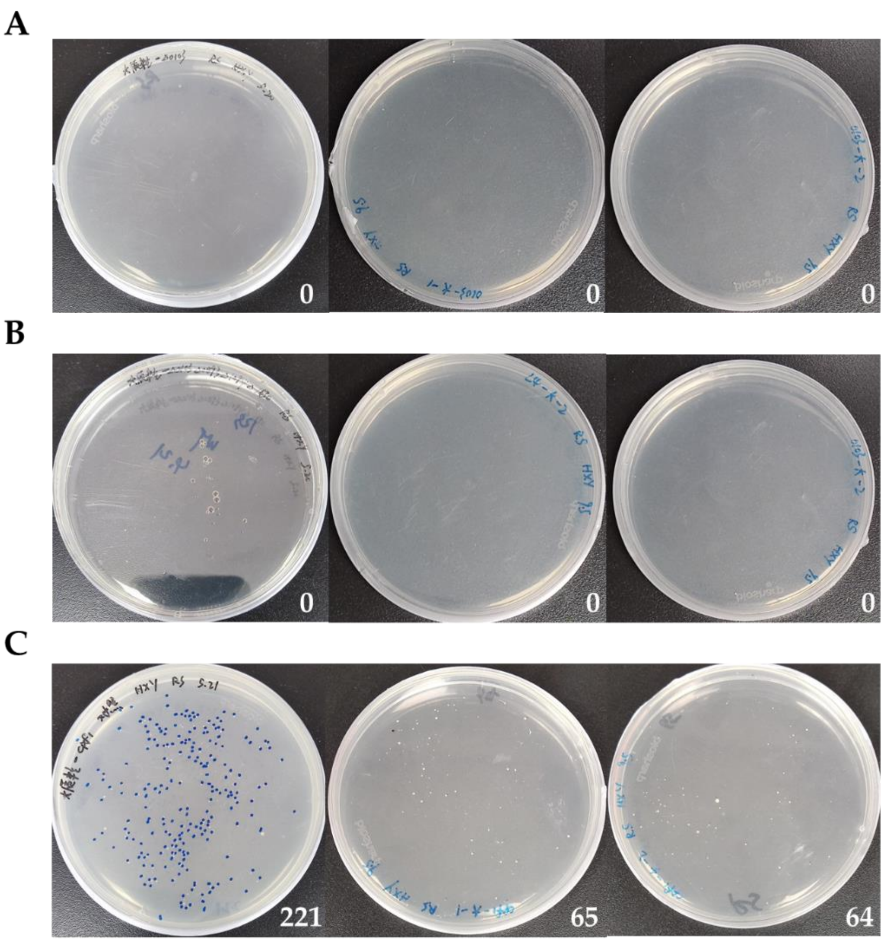

Figure S6. Electroporation efficiency of ZM4Δ0103, ZM4ΔARs and ZM4 by pEZ15A and pE39-MVA. Electroporation efficiency was tested by transforming pE39-MVA into ZM4Δ0103 (A) and ZM4ΔARs (B), whereas transformed into ZM4 (C) as control. An equal volume of transformants was spread onto RM plates containing to 100 μg/mL spectinomycin. Numbers in the lower right corner of each plate represent colony numbers. Three replicates were performed for each Electroporation.

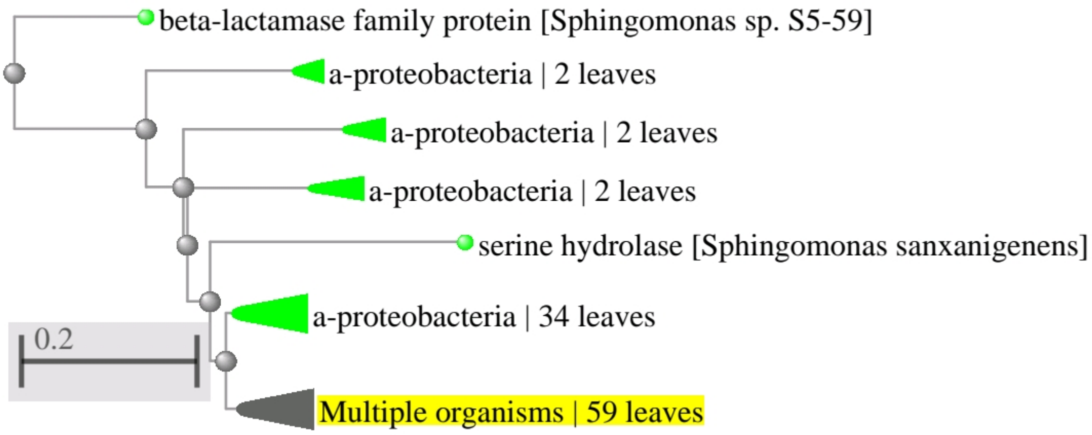

Figure S7. Distance tree of ZMO0103 after multiple sequence alignment with other proteins collected in NCBI using BALSTP.

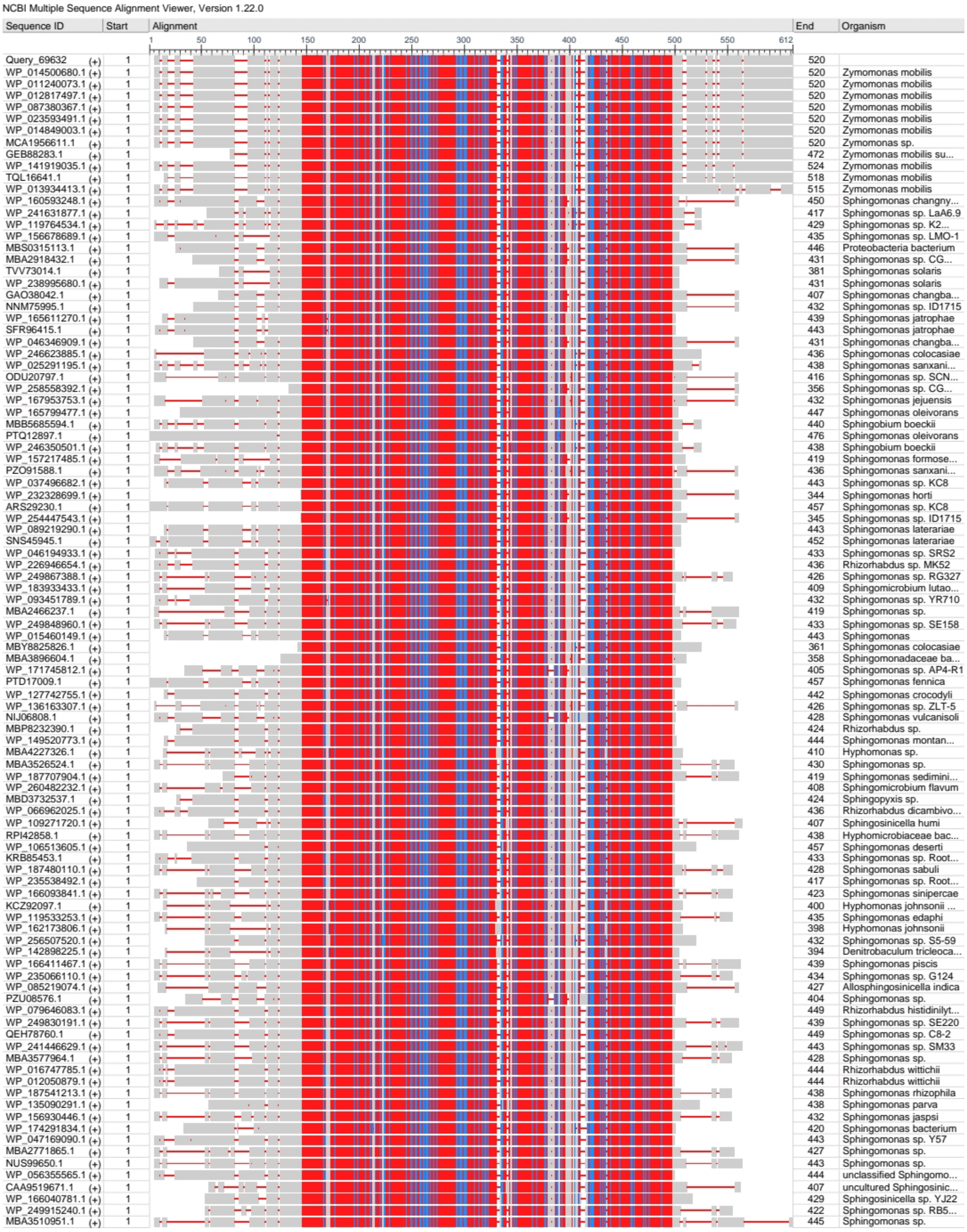

Figure S8. The detailed result of multiple sequence alignment with other proteins collected in NCBI using BALSTP.
