# Supplementary material for: Identification and Characterization of Genes Related to Ampicillin Antibiotic Resistance in Zymomonas mobilis
Source: Antibiotics (Basel). 2022 Oct 25;11(11):1476. doi: 10.3390/antibiotics11111476 (PMC9686808; doi:10.3390/antibiotics11111476)
Supplement: Supplementary file 1 [file antibiotics-11-01476-s001.zip › Supplementary Tables S3-S5.pdf]

Table S3. Strains used in this study.

Strains	Relevant characteristics	Source
**Strains for shuttle plasmids**		
DH5α	*E. coli* for plasmid construction	Lab stock
**Strains for genome-editing based on CRISPR-Cas12a system**		
ZM4	*Z. mobilis* subsp. *mobilis* ZM4 (wild-type strain)	Lab stock
ZM4-Cas12a	The Cas12a expressing cassette with Spe resistance gene replaced *ZMO0038*	Lab stock
ZM4Δ0103	ZM4 with deletion of *ZMO0103*	This study
ZM4Δ0893	ZM4 with deletion of *ZMO0893*	This study
ZM4Δ1650	ZM4 with deletion of *ZMO1650*	This study
ZM4Δ1866	ZM4 with deletion of *ZMO1866*	This study
ZM4ΔARs	ZM4 with deletions of *ZMO0103/0893/1094/1650*	This study

Table S4. Plasmids used in this study.

Plasmids	Relevant characteristics	Source
**Plasmids for genome-editing based on CRISPR-Cas12a system**		
pEZ-sgr-0103	Plasmid contains an artificial mini-CRISPR cluster based on CRISPR-Cas12a system and targets genome *ZMO0103*; Cm^R	This study
pEZ-sgr-0893	Plasmid contains an artificial mini-CRISPR cluster based on CRISPR-Cas12a system and targets genome *ZMO0893*; Cm^R	This study
pEZ-sgr-1094	Plasmid contains an artificial mini-CRISPR cluster based on CRISPR-Cas12a system and targets genome *ZMO1094*; Cm^R	This study
pEZ-sgr-1650	Plasmid contains an artificial mini-CRISPR cluster based on CRISPR-Cas12a system and targets genome *ZMO1650*; Cm^R	This study
pEZ-sgr-1866	Plasmid contains an artificial mini-CRISPR cluster based on CRISPR-Cas12a system and targets genome *ZMO1866*; Cm^R	This study
pEZ-sgr-1967	Plasmid contains an artificial mini-CRISPR cluster based on CRISPR-Cas12a system and targets genome *ZMO1967*; Cm^R	This study
pEZ-sgr-0103-D	Plasmid contains an artificial mini-CRISPR cluster based on CRISPR-Cas12a system and a donor to target genome	This study

	ZMO0103; Cm^R	
pEZ-sgr-0893-D	Plasmid contains an artificial mini-CRISPR cluster based on CRISPR-Cas12a system and a donor to target genome *ZMO0893*; Cm^R	This study
pEZ-sgr-1094-D	Plasmid contains an artificial mini-CRISPR cluster based on CRISPR-Cas12a system and a donor to target genome *ZMO1094*; Cm^R	This study
pEZ-sgr-1650-D	Plasmid contains an artificial mini-CRISPR cluster based on CRISPR-Cas12a system and a donor to target genome *ZMO1650*; Cm^R	This study
pEZ-sgr-1866-D	Plasmid contains an artificial mini-CRISPR cluster based on CRISPR-Cas12a system and a donor to target genome *ZMO1866*; Cm^R	This study
pEZ-sgr-1967-D	Plasmid contains an artificial mini-CRISPR cluster based on CRISPR-Cas12a system and a donor to target genome *ZMO1967*; Cm^R	This study
pEZ15A	Shuttle vector contains *Z. mobilis* origin and *E. coli* origin 15A; Sp^R	Lab stock
pE39-MVA	Shuttle vector contains *Z. mobilis* origin, *E. coli* origin 15A and MVA pathway genes	Lab stock

Table S5. Primers used in this study.

Primer names	Primer sequence (5′ to 3′)
**Primers for genome-editing based on CRISPR-Cas12a system**	
pEZ-SgR-0103-F	AGATCCGGATCCCAGCCGCTGGTGGTG
pEZ-SgR-0103-R	TGACCACCACCAGCGGCTGGGATCCGG
pEZ-SgR-0893-F	AGATTGGGTAAGGCAATCTCTGAAAGT
pEZ-SgR-0893-R	TGACACTTTCAGAGATTGCCTTACCCA
pEZ-SgR-1094-F	AGATAACAACATCACGGATGAAATGGC
pEZ-SgR-1094-R	TGACGCCATTTCATCCGTGATGTTGTT
pEZ-SgR-1650-F	AGATCCAATACTTCTTTTGAAGGGATG
pEZ-SgR-1650-R	TGACCATCCCTTCAAAAGAAGTATTGG
pEZ-SgR-1866-F	AGATCTTGCACCAGACTGCGCGGAATA
pEZ-SgR-1866-R	TGACTATTCCGCGCAGTCTGGTGCAAG
0103-US-F	GATGCCTGGAGATCCTTACTTCATGGAGCGTCCATCG
0103-US-R	GGCCTCGCTTTGCCTTTAGACAGTCACAACAAAGGGG
0103-DS-F	AGGCAAAGCGAGGCCTTTC

0103-DS-R	TAAATAAGGATCCAAACTCGCGACATATTGGAAACGGTCAG
0893-US-F	GATGCCTGGAGATCCTTACTCCGAAGATGTGTTCAAATCTGC
0893-US-R	TTATTCCAAGCGGCATTTTTCTG
0893-DS-F	AGAAAAATGCCGCTTGGAATAACTGCCTTTAATCTGTTCAGAAGC
0893-DS-R	TAAATAAGGATCCAAACTCGCAGAACCGCTTCTTCCCC
1094-US-F	GATGCCTGGAGATCCTTACTAAAGCAGCCAAGGATAAAGC
1094-US-R	CCCTGCCCGATTATTGATTTTATCGAACCGAGAAGACGGAG
1094-DS-F	ATAAAATCAATAATCGGGCAGGGG
1094-DS-R	TAAATAAGGATCCAAACTCGATACCCACTAAGGCACCAG
1650-US-F	GATGCCTGGAGATCCTTACTATCCGCCCGCCAATATTATG
1650-US-R	TTATCCAAAGGTTTTGGTGCG
1650-DS-F	CGCACCAAAACCTTTGGATAAGGTGCGGTCTTGATTAGCC
1650-DS-R	TAAATAAGGATCCAAACTCGCAATAACAGCGACGAAACCG
1866-US-F	GATGCCTGGAGATCCTTACTAACGCGGGCTCATCTTC
1866-US-R	GCAGATTTTAGCCCCCTGTAAGTGCATGTCTGATTTGGCTTTATC
1866-DS-F	CCTTGCAGCAGGCTTTAG

1866-DS-R	CTGGCCGGGTTCTAACC
0103-out-F	GATTGTCGAACGCGTAACCG
0103-out-R	GTCGATCAAGCCGTTGC
0893-out-F	ATCAGAGCAAGCCTGACCAC
0893-out-R	CCCAATCAGGCTTTGGATGC
1094-out-F	GCGTCCTATCTTTCATCGGC
1094-out-R	GAATAAAGATGCGGTTAGTTGCAG
1650-out-F	CTTTTGCCCTGCCCTAACG
1650-out-R	CGCTTGAGAACTTCCACCATC
1866-out-F	CCTTGCAGCAGGCTTTAG
1866-out-R	CTGGCCGGGTTCTAACC
pEZ15A-F	GGCAAAGCCACCCTATTTTTAG
pEZ15A-R	CACTTCACTGACACCCTCAT
cpf1-kf-F	CGAGTTTGGATCCTTATTTATACAATTCATCCATACC
cpf1-kf-R	AGTAAGGATCTCCAGGCATCAAATAAAACGA
